# Most bicarbonate secretion by Calu‐3 cells is mediated by CFTR and independent of pendrin

**DOI:** 10.14814/phy2.13641

**Published:** 2018-03-13

**Authors:** Junwei Huang, Dusik Kim, Jiajie Shan, Asmahan Abu‐Arish, Yishan Luo, John W. Hanrahan

**Affiliations:** ^1^ Department of Physiology McGill University Montréal Québec Canada; ^2^ Cystic Fibrosis Translational Research Center McGill University Montréal Québec Canada; ^3^ Research Institute‐McGill University Health Centre Montréal Québec Canada; ^4^ Present address: AbbVie Bioresearch Center AbbVie Inc. 381 Plantation St. Worcester MA 01605; ^5^ Present address: School of Medicine South China University of Technology Guangzhou University Town Panyu District Guangzhou China

**Keywords:** Airway epithelial cells, cystic fibrosis, pendrin, SLC26A4

## Abstract

Bicarbonate plays an important role in airway host defense, however, its transport mechanisms remain uncertain. Here we examined the relative contributions of the anion channel CFTR (cystic fibrosis transmembrane conductance regulator, ABCC7) and the anion exchanger pendrin (SLC26A4) to HCO_3_^−^ secretion by the human airway cell line Calu‐3. Pendrin and CFTR were both detected in parental Calu‐3 cells, although mRNA and protein expression appeared higher for CFTR than for pendrin. Targeting pendrin transcripts with lentiviral shRNA reduced pendrin detection by immunofluorescence staining but did not alter the rates of HCO_3_^−^ or fluid secretion, HCO_3_^−^ transport under pH‐stat conditions, or net HCO_3_^−^ flux across basolaterally permeabilized monolayers. Intracellular pH varied with step changes in apical Cl^−^ and HCO_3_^−^ concentrations in control and pendrin knockdown Calu‐3 cells, but not in CFTR deficient cells. Exposure to the proinflammatory cytokine IL‐4, which strongly upregulates pendrin expression in airway surface epithelia, had little effect on Calu‐3 pendrin expression and did not alter fluid or HCO_3_^−^ secretion. Similar results were obtained using air–liquid interface and submerged cultures, although CFTR and pendrin mRNA expression were both lower when cells were cultured under submerged conditions. While the conclusions cannot be extrapolated to other airway epithelia, the present results demonstrate that most HCO_3_^−^ secretion by Calu‐3 cells is mediated by CFTR.

## Introduction

The airways are lined by epithelia that secrete fluid and mucus which enable the clearance of inhaled substances. Fluid secretion is driven primarily by transepithelial Cl^−^ transport, a two‐step process in which basolateral Cl^−^ loading occurs by cotransport with sodium and potassium and also by exchange with bicarbonate, followed by Cl^−^ efflux through apical anion channels (Huang et al. 2012; Shan et al. 2012). HCO_3_^−^ is also secreted into the airway lumen (Smith and Welsh 1992) where it functions in mucin unpacking (Quinton 2008) and bacterial killing (Pezzulo et al. 2012). Despite the importance of bicarbonate secretion, the mechanisms of apical HCO_3_^−^ efflux remain uncertain.

cAMP‐stimulated anion secretion has been studied extensively using the model human epithelial cell line Calu‐3 as a model for serous cells of the submucosal glands, which mediate much of the secretion in the upper airways (Widdicombe and Wine 2015). Calu‐3 cells resemble gland cells in forming polarized monolayers with tight junctions and transepithelial resistance >100 Ω·cm^2^ at the air–liquid interface (Shen et al. 1994) but have fewer dense granules and lower antimicrobial peptide expression compared to gland serous cells. Nevertheless, Calu‐3 cells express the submucosal gland markers lysozyme (Duszyk 2001) and lactoferrin (Dubin et al. 2004), CFTR protein (Haws et al. 1994), and have robust cAMP‐stimulated short‐circuit currents (*I*_sc_) that are stimulated by *β*‐adrenergic agonists, vasoactive intestinal peptide, and adenosine (Shen et al. 1994).

CFTR conducts HCO_3_^−^ (Gray et al. 1989; Poulsen et al. 1994; Linsdell et al. 1997) and was proposed to mediate apical HCO_3_^−^ efflux from airway epithelial cells (Poulsen et al. 1994) including Calu‐3 (Lee et al. 1998; Devor et al. 1999; Shan et al. 2012). Estimates of the driving force for HCO_3_^−^ efflux and apical conductance suggest that HCO_3_^−^ electrodiffusion through CFTR would be sufficient to explain the net flux (Tamada et al. 2001), and some intracellular pH (pH_i_) and patch clamp data support CFTR‐mediated HCO_3_^−^ exit (Kim et al. 2014). However, there is also evidence for predominant Cl^−^/HCO_3_^−^ exchange mediated by pendrin (SLC26A4) in Calu‐3 cells (Garnett et al. 2011). Pendrin is an electroneutral exchanger that carries HCO_3_^−^, Cl^−^, OH^−^, Br^−^, I^−^, nitrate or thiocyanate (Pedemonte et al. 2007; Shcheynikov et al. 2008; Ohana et al. 2009; Alper and Sharma 2013) and is expressed in diverse tissues including the inner ear (Everett et al. 1999), thyroid gland (Everett et al. 1997), kidney (Scott et al. 1999; Royaux et al. 2000, 2001; Lacroix et al. 2001), mammary gland (Rillema and Hill 2003), testis (Lacroix et al. 2001), placenta (Bidart et al. 2000), endometrium (Suzuki et al. 2002), and liver (Alesutan et al. 2011).

The possible role of pendrin in the airways has generated much interest since it was shown to be elevated in murine models of asthma and chronic obstructive pulmonary disease (Kuperman et al. 2005). Pendrin expression is strongly upregulated when primary human bronchial epithelial (HBE) cells are exposed to the proinflammatory cytokines IL‐4/IL‐13 or IL‐17A (Pedemonte et al. 2007; Nakagami et al. 2008; Nofziger et al. 2011; Adams et al. 2014). Elevated pendrin expression in nasal cells in vitro is accompanied by more rapid intracellular alkalinization when cells are challenged with apical low‐Cl^−^ solution, consistent with enhanced HCO_3_^−^/Cl^−^ exchange (Widdicombe and Wine 2015). Moreover, the lack of pendrin in cells from people with pendred syndrome and from pendrin‐null mice leads to an increase in airway surface liquid (ASL) height, suggesting that pendrin inhibitors could be useful therapeutics for increasing airway hydration in CF (Lee et al. 2015; Haggie et al., 2016).

Our goal was to examine the possible roles of pendrin and CFTR in HCO_3_^−^ secretion by Calu‐3 cells. We studied the contribution of pendrin to fluid and HCO_3_^−^ secretion by control and pendrin knockdown Calu‐3 cell lines. We also investigated the effects of IL‐4 and culture conditions (air–liquid interface vs. submerged) on pendrin functional expression in Calu‐3.

## Materials and Methods

### Calu‐3‐derived cell lines

Calu‐3 cells from American Type Culture Collection (ATCC, Rockville, MD) were cultured in Eagle's Minimum Essential Medium (Wisent Bioproducts Inc., Saint‐Jean‐Baptiste, QC). Basal medium contained NaHCO_3_, essential amino acids, and 2 mmol/L glutamine and was supplemented with 1% MEM nonessential amino acids (Wisent), 1% sodium pyruvate (Sigma, St. Louis MO) as recommended by ATCC (Huang et al. 2012; Kim et al. 2014), and with 7% fetal bovine serum (FBS; Wisent). Growth rates, electrophysiological properties, and mRNA expression for transporters including CFTR and pendrin were similar when media contained 7% or 10% FBS (data not shown). Five pendrin knockdown Calu‐3 cell lines (PDS‐KD) were generated from the parental line using shRNA lentiviral vectors (see Table 1; Open Biosystems, Lafayette, CO) that target pendrin distal to nucleotide 1296, a region conserved between the two known splice variants (http://www.uniprot.org/uniprot/O43511). Each transfer vector was cotransfected into HEK293T cells together with packaging (psPAX2) and envelope (pMD2.G) plasmids and the resulting lentiviral particles were used to transduce Calu‐3 cells. Puromycin (4 *μ*g·mL^−1^; Wisent) was added 48 h after transduction. Resistant colonies were amplified and transferred at ~10^6^ cells·cm^−2^ onto Snapwell™ filters (1.12 cm^2^, 0.4 *μ*m pores, polycarbonate, Corning Life Science, Nepean, ON) for electrophysiology and imaging experiments, or onto Transwells™ (4.67 cm^2^, Corning) for fluid secretion assays. A control cell line expressing scrambled shRNA (Scr‐KD; Addgene, Cambridge, MA; Plasmid ID: #1864) was generated using the same procedures. A CFTR knock down cell line (CFTR‐KD) stably expressing shRNA was kindly provided by Dr. Scott O'Grady (Univ. Minnesota) and has been characterized previously (Palmer et al. 2006b; Shan et al. 2012). In most experiments, apical medium was removed after 24–48 h to establish an air–liquid interface (ALI) and basolateral medium was replaced at 2 day intervals. To study submerged cultures, ~200 *μ*L medium was maintained on the apical surface. Transepithelial resistance was monitored using an epithelial volt‐ohmmeter (World Precision Instr., Sarasota, FL). Monolayers were used 10–28 days after plating when the resistance had reached a plateau of ~400 Ω·cm^2^.

**Table 1:** shRNA sequences used for lentiviral knockdown of pendrin in Calu‐3 cells

TRCN0000044283	CCGGGCGATTGTGATGATCGCCATTCTCGAGAATGGCGATCATCACAATCGCTTTTTG
TRCN0000044284	CCGGCCAGCAGCAATGGAACTGTATCTCGAGATACAGTTCCATTGCTGCTGGTTTTTG
TRCN0000044285	CCGGCCAACCTGAAAGGGATGTTTACTCGAGTAAACATCCCTTTCAGGTTGGTTTTTG
TRCN0000044286	CCGGGCTATATCTTTCCTGGACGTTCTCGAGAACGTCCAGGAAAGATATAGCTTTTTG
TRCN0000044287	CCGGCCCTATCCTGACATACTTTATCTCGAGATAAAGTATGTCAGGATAGGGTTTTTG

### Quantitative real‐time PCR

Total RNA was isolated from ALI cultures using the RNeasy Mini Kit (Qiagen) following the manufacturer's instructions. First‐strand cDNA for real‐time PCR was generated using 1 *μ*g of total RNA and SuperScript VILO MasterMix (Invitrogen). The levels of CFTR, SLC26A4, SLC26A6, and SLC26A9 mRNA were assessed by Quantitative real‐time PCR (qRT‐PCR). Taqman primers (Life technologies; Assay ID: CFTR: Hs00357011_m1; SLC26A4: Hs01070620_m1; SLC26A6: Hs00370470_m1; SLC26A9: Hs00369451_m1) and the QuantStudio™ 7 Flex Real‐Time PCR system (Life Technologies) were used. qRT‐PCR was performed using the “Fast” program: 95°C for 20 sec, followed by 40 cycles of 95°C for 1 sec and 60°C for 20 sec. Data were normalized to GAPDH.

### Immunoblotting

Cells were solubilized in RIPA buffer (150 mmol·L^−1^ NaCl, 1 mmol·L^−1^ Tris/HCl, 1% deoxycholic acid, 1%Triton X‐100, 0.1% SDS and protease inhibitor) for 15–30 min on ice and centrifuged at 32,000*g* for 10 min at 4°C. Supernatant was collected and assayed for total protein concentration (Bio‐Rad). Equivalent amounts of protein from each sample were run on 10% SDS‐PAGE gels and transferred to polyvinylidene difluoride (PVDF) membranes for immunoblotting. PVDF membranes were blocked with 5% nonfat dried skimmed milk in TTBS [(Tris‐buffered saline; 50 mmol·L^−1^ Tris and 150 mmol·L^−1^ NaCl, pH 8.0) supplemented with 0.2% Tween 20] for at least 1 h, then incubated with primary antibodies in TBS overnight at 4°C. The rabbit antipendrin polyclonal antibody PN826 was kindly provided by Dr. A. Griffith, NIDCD Bethesda MD (Choi et al. 2011). Rabbit polyclonal antibody against the COOH‐terminal amino acids 1224–1237 of mouse AE2 (SA6) was a generous gift of Dr. S. Alper, Beth Israel Deaconess Medical Center and Harvard Univ. The mouse monoclonal anti‐CFTR antibody (23C5, 1:50) was generated in collaboration with Dr. D.Y. Thomas, McGill Univ. The rabbit polyclonal anti‐NBC antibody AB3212 (1:500) was from Millipore. Anti‐NKCC1 (goat polyclonal, SC‐21545, 1:200) and goat polyclonal anti‐*β*‐actin antibody SC‐1615 (1:200) were from Santa Cruz Biotechnology. The Na^+^/K^+^‐ATPase *α* subunit (mouse monoclonal a5, 1:200) was a kind gift from Dr. R.W. Mercer, Washington Univ., St. Louis MO). Membranes were washed with TTBS, incubated with secondary antibody conjugated to horseradish peroxidase, and developed for enhanced chemiluminescence (Amersham Biosciences). Protein bands were analyzed by densitometry using EZQuant‐gel software (EZQuant, Israel).

### Immunocytochemistry

**Figure 1:**
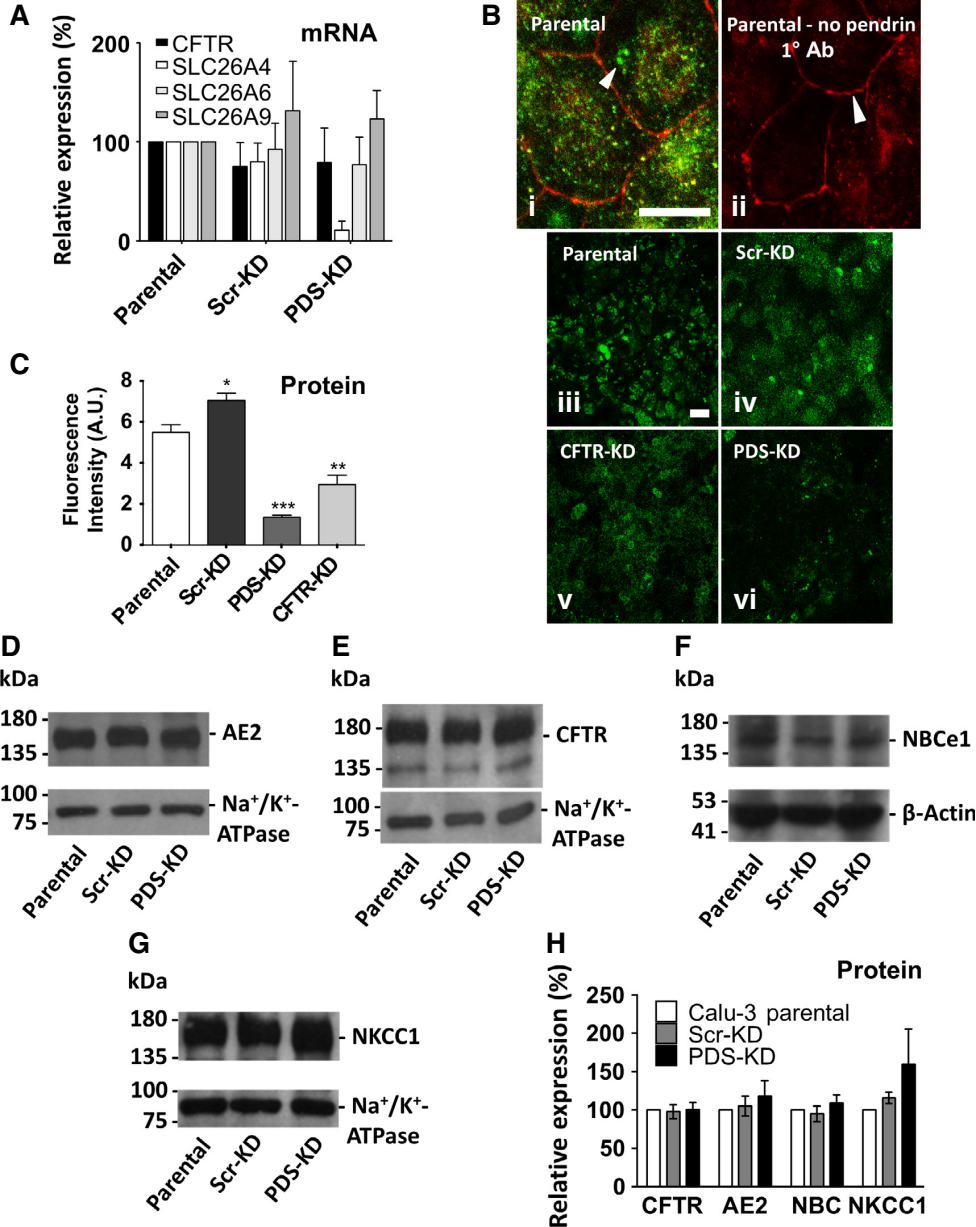
Pendrin expression and generation of pendrin knockdown (PDS‐KD) Calu‐3 cell lines. (A) CFTR, SLC26A4, SLC26A6, and SLC26A9 mRNA expression in Scr‐KD (control) and PDS‐KD (pendrin knockdown) cells. mRNA levels were measured using qRT‐PCR, normalized to GAPDH, and plotted relative to the corresponding value in parental Calu‐3 cells. PDS mRNA expression was unchanged in Scr‐KD cells and was reduced ~90% in the PDS‐KD line transduced with sh‐PDS‐5 (*n* = 5, *P* < 0.0001). CFTR, SCL26A6, and A9 mRNA levels were not altered significantly in pendrin knockdown cells. (B) (i) immunstaining ZO‐1 (red) and pendrin (green) in parental Calu‐3 (WT) cells, (ii) same as (i) but omitting antipendrin primary antibody, panels (iii)–(vi) pendrin immunostaining in WT cells, scrambled control shRNA cells, CFTR knockdown cells, and pendrin knockdown cells, respectively. All images were taken using the same illumination intensity and laser power. Scale bars: 20 *μ*m. Images are representative of *n* = 4–5 cultures/cell line. (C) summary of pendrin fluorescence intensity in parental (WT), Scr‐KD (shRNA scrambled control cells), PDS‐KD (pendrin knockdown cells) and CFTR‐KD cells (CFTR knockdown cells, *n* = 4–5, ± SEM). Pendrin staining was reduced ~80% in PDS‐KD compared to parental and scrambled shRNA control cells. Unpaired Student's *t* tests show *P <* *0.05, **P <* *0.01, ***P <* *0.001. (D–G) immunoblots of Calu‐3 parental, Scr‐KD, and PDS‐KD cells. 20 *μ*g total protein was probed with antibody against: (D) AE2 and Na^+^/K^+^‐ATPase *α*‐subunit; (E) CFTR and Na^+^/K^+^‐ATPase a‐subunit; (F) NBCe1 and *β*‐actin, and (G) NKCC1 and Na^+^/K^+^‐ATPase *α*‐subunit. (H) summary of expression of different transporters in parental, scrambled shRNA control, and pendrin knockdown cell lines. Each protein was corrected for loading using *β*‐actin or Na^+^/K^+^‐ATPase, and normalized to the expression in parental Calu‐3 cells.

Cells cultured on Transwells were washed with PBS three times to remove apical secretions and fixed with 10% neutral‐buffered formalin for 15 min at RT. After washing with PBS again, cells were permeabilized with 1% Triton X‐100 then blocked with 2% BSA for 1 h. When staining for pendrin alone, samples were incubated with rabbit polyclonal antipendrin antibody (H‐195; Santa Cruz) at 1:500–1:1000 dilution overnight at 4°C, followed by goat anti‐rabbit IgG Alexa fluor 488 secondary antibody (Invitrogen, 1:1000). When staining both pendrin and ZO‐1, goat polyclonal antipendrin (E20; 1:500; Santa Cruz) was used followed by donkey anti‐goat IgG Alexafluor 488, 1:1000; Invitrogen) for 1 h at RT. ZO‐1 was immunostained using rabbit anti‐ZO‐1 antibody (Life technology; 1:1000) followed by goat anti‐rabbit Alexa 594 (Invitrogen; 1:1000) secondary antibody for 1 h at RT. Some samples were also exposed for 1 min to the nuclear stain DAPI (1 *μ*g·mL^−1^). The absence of staining when the primary antibody was omitted served as a negative control. Cell imaging was performed using an LSM‐780 confocal microscope (Zeiss, Germany), equipped with a multiline argon laser (488 nm, 25 mW) for Alexa‐488 excitation and a 560 nm line laser (15 mW) for Alexa‐594 excitation. Imaging was performed using a Plan‐Apochromat 63× oil immersion objective and digital gain setting of 900. Brief exposures to 5% laser power were used to maximize detection of pendrin in Calu‐3 cells and 2% laser power used when quantifying expression. Imaging conditions were optimized to avoid pixel saturation. Relative fluorescence intensity measurements (arbitrary units [A.U.]) were analyzed using ImageJ (e.g., Fig. 1, (Rasband 2011)) or MetaXpress software (Molecular Devices, Sunnyvale CA).

### Fluid secretion

Apical fluid that was present initially was removed and 1/1000 DMSO control (v/v) or 10 *μ*mol·L^−1^ forskolin + 200 *μ*mol·L^−1^ cAMP was added to the basolateral side. A microelectrode (9826BN, Orion) was used to measure the pH of secretions in humidified 5% CO_2_/95% air at 37°C. The volume of the fluid measured at 24 h intervals by aspiration yielded reproducible measurements of cAMP‐stimulated fluid secretion.

### Apical anion exchange assayed in Ussing chambers using pH‐stat

Snapwell^®^ filters were mounted in Ussing chambers and current‐clamped using a VCCMC6 amplifier (EasyMount, Physiologic Instruments, San Diego CA). Basolateral Krebs–Henseleit solution contained (in mmol·L^−1^) 120 NaCl, 25 NaHCO_3_, 3.3 KH_2_PO_4_, 0.8 K_2_HPO_4_, 1.2 CaCl_2_, 1.2 MgCl_2_, and 10 glucose, and was gassed with 95% O_2_/5% CO_2_. Apical pH‐stat solution contained (in mmol·L^−1^): 150 NaCl, 1.2 CaCl_2_, 1.2 MgCl_2_, and 5 KCl and was bubbled with 100% O_2_. pH‐stat experiments were performed at 37 °C. Equivalent short‐circuit current (*I*_eq_) was calculated at 100 sec intervals using the spontaneous transepithelial potential (V_t_) and the resistance (R_t_), which was determined by injecting small constant‐current pulses (1 *μ*A, 1 sec duration). A mini‐pH electrode (pHG200‐8, Radiometer Analytical) connected to a titration workstation (TitraLab 854, Radiometer) delivered 1 *μ*L aliquots of 10 mequiv·L^−1^ HCl automatically to maintain the pH at 7.000 ± 0.002 and the volume of HCl was used to calculate net HCO_3_^−^ secretion. To assay apical ion exchange, Cl^−^ was replaced bilaterally with gluconate, the basolateral membrane was permeabilized for 15 min using nystatin (360 *μ*g·mL^−1^), and either NaCl or NaI solution was added to the apical side to create a 30 mmol/L apical‐to‐basolateral gradient for Cl^−^ or I^−^, respectively (Shan et al. 2012). The secretory flux of HCO_3_^−^ was monitored by apical pH‐stat as described above.

### Intracellular pH (pH_i_) assays of anion exchange

Intracellular pH was measured using an inverted fluorescence microscope (IX81, Olympus, Center Valley PA) and imaging system (Photon Technology International, Edison NJ). Confluent cell monolayers on inserts were loaded with the pH‐sensitive dye BCECF (2′,7′‐*bis*‐(2‐carboxyethyl)‐5‐(and 6)‐carboxyfluorescein by removing the medium, washing for 30 min in HCO_3_^−^‐buffered solution, then incubating cells with 5 *μ*mol·L^−1^ BCECF‐AM in HCO_3_^−^‐buffered 5% CO_2_ solution at 37°C for ≥45 min. Cells were then rinsed and mounted in a chamber that allowed independent perfusion of the apical and basolateral surfaces. Flow was maintained at 1.5 mL·min^−1^ using a four‐channel peristaltic pump (205S, Watson Marlow, Wilmington MA), which also removed solutions by suction. The temperature was maintained at 37°C using a thermostatically controlled platform (FC‐5, Live Cell Instr., Seoul, Korea).

Apical and basolateral sides were initially perfused with HCO_3_^−^‐buffered solution for 5–10 min or until pH_i_ became stable. The HCO_3_^−^ solution contained (mmol·L^−1^): 116 NaCl, 25 Na HCO_3_^−^, 5 KCl, 1 CaCl_2_, 1 MgSO_4_, 2.8 NaHEPES, 2.2 HEPES, and 10 D‐glucose. Changes in pH_i_ were measured during brief exposure of the apical or basolateral sides to nominally Cl^−^‐free solution containing (in mmol·L^−1^): 116 sodium gluconate, 25 Na HCO_3_^−^, 5 potassium gluconate, 4 calcium gluconate, 1 MgSO_4_, 2.8 NaHEPES, 2.2 HEPES, and 10 D‐glucose. When apical [HCO_3_^−^] was increased 2‐fold, the high‐ HCO_3_^−^ solution contained (mmol·L^−1^): 100 NaCl, 50 Na HCO_3_^−^, 1 MgSO_4_, 2.8 NaHEPES, 2.2 HEPES, and 10 D‐glucose and pH was allowed to increase to 7.8, where it remained for >1 h when bubbled with 95% O_2_/5% CO_2_. Normal HCO_3_^−^ solution contained 100 NaCl, 25 Na HCO_3_^−^, 25 sodium gluconate, 1 MgSO_4_, 2.8 NaHEPES, 2.2 HEPES, and 10 D‐glucose and remained at pH = 7.5 when bubbled with 95% O_2_/5% CO_2_. When HCO_3_^−^ concentration was reduced by half, the low‐ HCO_3_^−^ solution contained: 100 NaCl, 12.5 Na HCO_3_^−^, 37.5 sodium gluconate, 1 MgSO_4_, 2.8 NaHEPES, 2.2 HEPES, and 10 D‐glucose and the pH was allowed to decrease to ~7.2, where it remained for the duration of the experiment when bubbled with 95% O_2_/5% CO_2_. Bicarbonate was not directly measured therefore the calculated concentrations are considered estimates. All solutions were maintained at 37°C.

BCECF was excited alternately at 440 and 490 nm for 1 s at each wavelength, with 5 s between each pair of measurements. Since individual cells could not be resolved in the confluent monolayers, unbiased sampling of pH_i_ was performed as follows: Fluorescence was measured in five regions of interest (ROIs) per field, one at the outer edge of each of four quadrants and one near the center. These ROIs covered ∼2/3 of the total area in the field of view using a 40× objective. Fluorescence intensities in the five ROIs were averaged to obtain one value. Fluorescence ratios (*F*490/*F*440) were recorded and displayed continuously using Easy Ratio Pro software. To calculate pH_i_, *F*490/*F*440 was calibrated using high‐K^+^‐nigericin solutions containing (mmol·L^−1^): 140 KCl, 1 CaCl_2_, 1 MgSO_4_, 20 HEPES, and 20 *μ*mol·L^−1^ nigericin, adjusted to different pH values. Graphs and statistical analyses were performed using Microsoft Excel and Prism 5 software.

### Statistics

Data are means ± standard error of mean (SEM) of *n* observations. Datasets were compared using the Student's *t* test or two‐way analysis of variance (GraphPad Prism) with *P* < 0.05 considered significant unless otherwise indicated.

## Results

### Generation of pendrin knockdown and scrambled control Calu‐3 cells

We began by confirming that pendrin and CFTR are expressed in Calu‐3 cells. Transcripts encoding CFTR, SLC26A4 (pendrin) and the related exchangers SLC26A6 and SLC26A9 were readily detected in lysates of parental Calu‐3 cells from ATCC (Fig. 1A). Transcript levels were normalized to those of GAPDH before determining relative levels in transduced versus parental cell lines. Control cells transduced with scrambled shRNA (Scr‐KD) did not have significantly altered mRNA levels for the four transporters examined. Lentiviral transduction of parental Calu‐3 cells with shRNAs targeting five different pendrin sequences (see Table 1) identified one sequence (sh‐PDS‐5) that greatly reduced PDS mRNA (to 10.7 ± 9%, mean ± SEM, *n* = 5, *P* < 0.0001; Fig. 1A). All experiments were performed using low‐passage, nonclonal cells transduced with sh‐PDS‐5 to minimize selection of atypical variants. Pendrin protein was not detected in parental Calu‐3 cell lysates by immunoblotting using several different antipendrin antibodies although they recognized heterologous pendrin overexpressed in HEK cells, thus it was not possible to convincingly detect pendrin protein in Calu‐3 by immunoblotting, as in previous studies (Garnett et al. 2011). Nevertheless, PDS knock down was confirmed at the protein level by immunostaining (green) and confocal imaging with settings identical to those used when imaging immunstained pendrin in Parental and Scr‐KD cells (Fig. 1B). Tight junctions were also visualized by immunostaining ZO‐1 (red). Pendrin was detected by immunostaining and was absent when the primary antipendrin antibody was omitted. Confocal images confirmed pendrin protein expression in WT, Scr‐KD and CFTR‐KD cells, which was reduced by 80–85% in PDS‐KD cells (Fig. 1B and C), similar to the mRNA decrease observed using qPCR (~90%; Fig. 1A). Interestingly, pendrin expression was also reduced in a CFTR knockdown cell line, suggesting there is a positive interaction between these proteins (Fig. 1B and C). Pendrin knockdown did not alter AE2, NBCe1, NKCC1, or CFTR expression by immunoblotting (Fig. 1D–H). Similar results were obtained whether *α*‐tubulin, Na^+^/K^+^‐ATPase or *β*‐actin was used as the loading control. Pendrin knockdown remained constant throughout the experiments according to qPCR performed at the beginning and end of the project.

### Volume and pH of cAMP‐stimulated secretions

**Figure 2:**
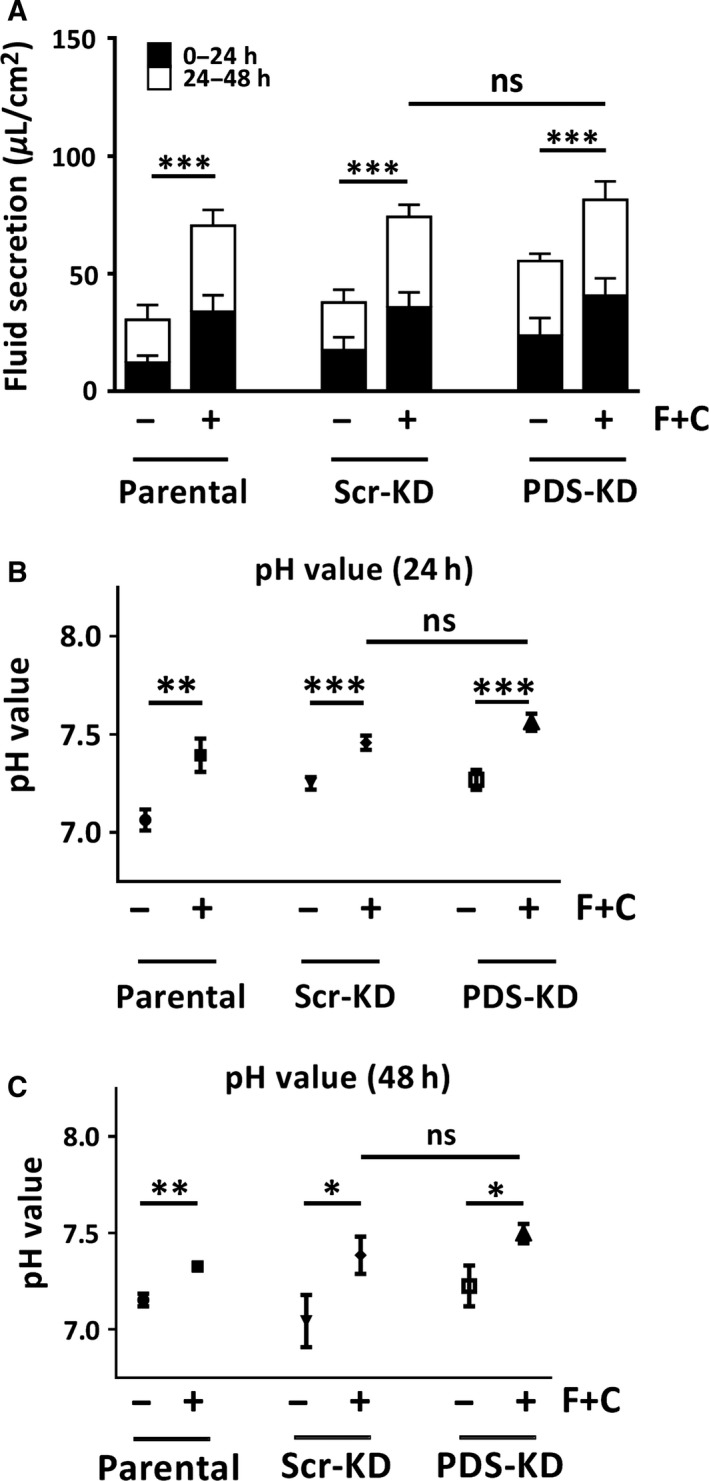
Fluid secretion volume and pH are similar in control and PDS‐KD Calu‐3 cells. Air–liquid interface cultures were exposed to basolateral DMSO vehicle or cAMP + forskolin (C + F). Apical fluid was collected at 24 h intervals for 2 days and plotted cumulatively. Apical pH was measured using a mini‐pH electrode. (A) fluid volume secreted during first and second day. (B and C) pH of the apical fluid after 24 and 48 h, respectively. Means ± SEM, *P < 0.1, **P < 0.05, ***P < 0.01.

To examine pendrin function we began by measuring fluid secretion volume and pH when parental, Scr‐KD and PDS‐KD monolayers were stimulated using cpt‐cAMP (200 *μ*mol·L^−1^) + forskolin (10 *μ*mol·L^−1^). Fluid was collected and measured after 24 and 48 h to assay fluid secretion while maintaining cells at the ALI. Figure 2A shows the cumulative fluid volumes. Forskolin + cpt‐cAMP caused similar increases in fluid secretion during the first and second days (parental: 76 ± 12 *μ*L·cm^−2^; Scr‐KD: 74 ± 11 *μ*L·cm^−2^; PDS‐KD: 88 ± 14 *μ*L·cm^−2^, *n* = 6–9, Scr‐KD, and PDS‐KD not different, *P* > 0.2). The pH of secretions was consistently higher during cAMP stimulation (Fig. 2B and C) and the alkalinization was similar for all three cell lines. These results suggest pendrin knockdown in Calu‐3 cells has little impact on basal or cAMP‐stimulated secretion of fluid or HCO_3_^−^.

### Forskolin‐stimulated *I*_eq_ and net HCO_3_^−^ flux under pH‐stat conditions

**Figure 3:**
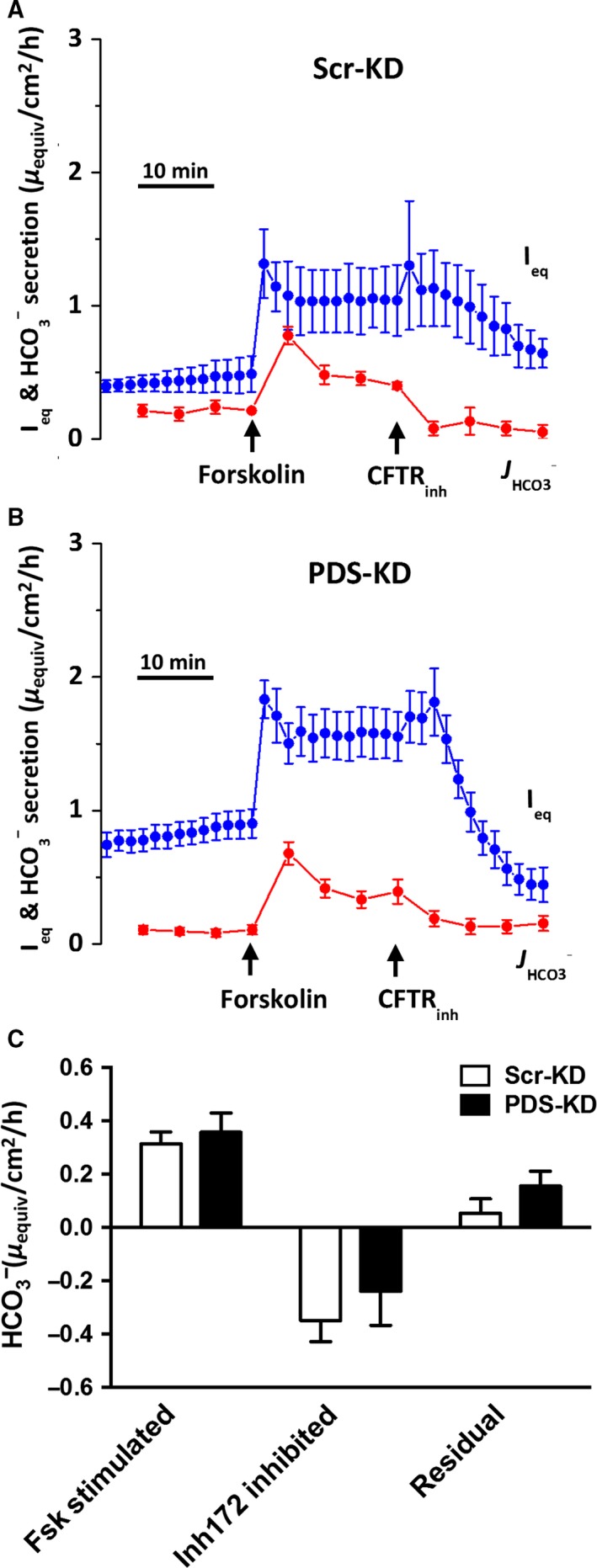
Pendrin knockdown does not affect forskolin‐stimulated HCO_3_^−^ secretion under open‐circuit, pH stat conditions. Forskolin and CFTR_Inh_‐172 were added sequentially to the basolateral and apical sides, respectively. *I*_eq_ (blue symbols) and HCO_3_^−^ secretion (red symbols) were monitored across (A) Scr‐KD, and (B) PDS‐KD monolayers. (C) net HCO_3_^−^ secretion rates. There was no difference in forskolin‐stimulated or residual HCO_3_^−^‐secretion rates after CFTR_Inh_‐172 when comparing Scr‐KD and PDS‐KD cells (mean ± SEM, *n* = 4–9; *P < 0.05).

Net HCO_3_^−^ secretion and electrogenic anion transport were monitored in Ussing chambers under open‐circuit, pH‐stat conditions (Huang et al. 2012; Shan et al. 2012). Equivalent short‐circuit current (*I*_eq_; blue symbols) and net HCO_3_^−^ flux (red symbols) time courses are shown for Scr‐KD (Fig. 3A) and PDS‐KD monolayers (Fig. 3B). Steady‐state HCO_3_^−^ transport rates estimated by pH‐stat are summarized in Figure 3C. Forskolin stimulated net HCO_3_^−^ fluxes of ~0.4 *μ*equiv·cm^−2^·h^−1^ in both Scr‐KD and PDS‐KD monolayers, and these fluxes were abolished by CFTR_inh_‐172. These results suggest that forskolin‐stimulated HCO_3_^−^ transport is CFTR‐dependent and unaffected by pendrin knockdown, consistent with the results described above when Calu‐3 cells were bathed apically with their own secretions.

### Apical Cl^−^/HCO_3_^−^ exchange in basolaterally permeabilized monolayers

**Figure 4:**
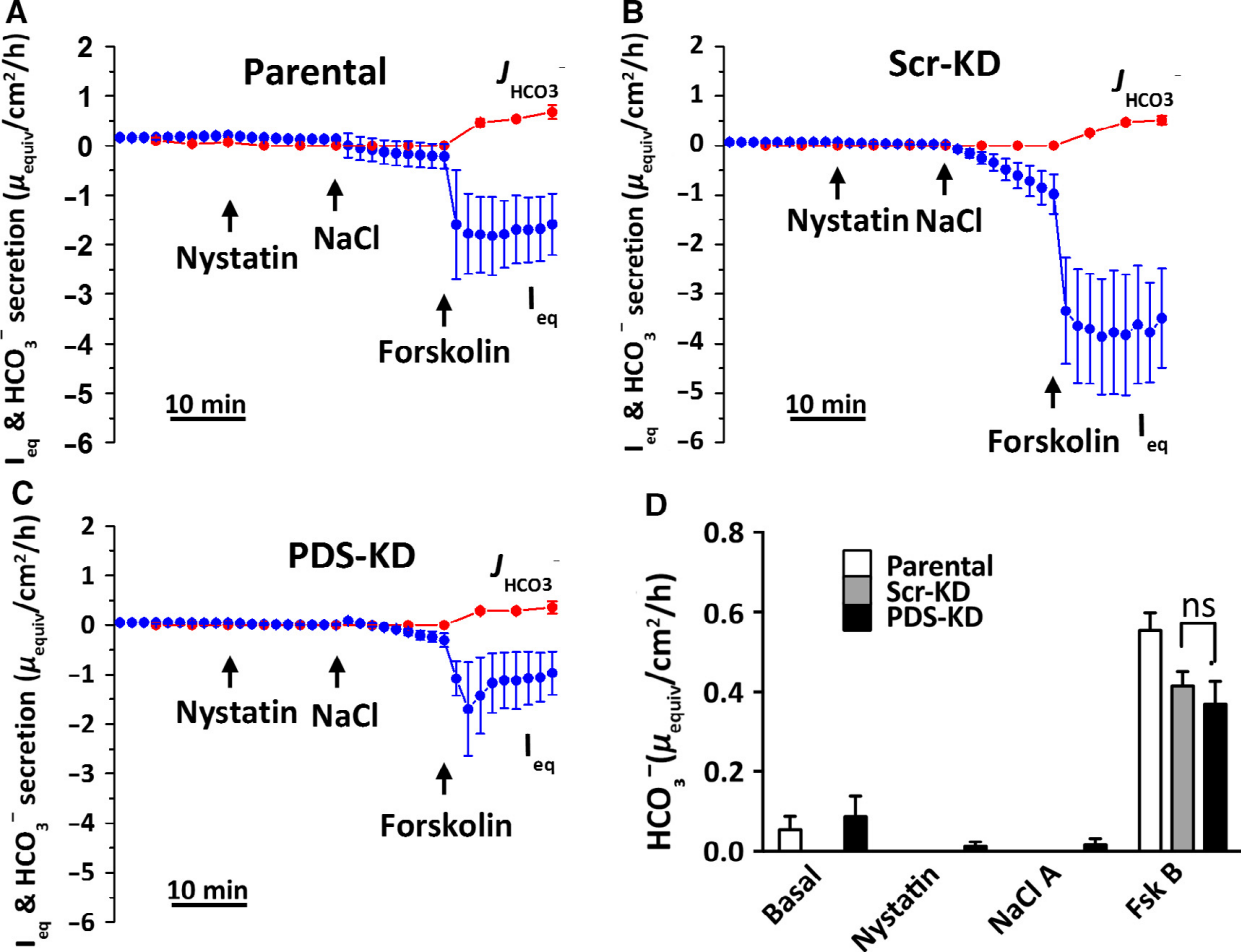
No evidence for pendrin‐dependent, apical Cl^−^/HCO_3_^−^ exchange in basolaterally permeabilized Calu‐3 monolayers. (A–C) cultures were bathed initially in symmetrical Cl^−^‐free solutions and permeabilized basolaterally using nystatin (360 *μ*g·mL^−1^). Apical HCO_3_^−^ efflux was monitored using pH stat. 30 mmol/L NaCl was added on the apical side to establish an apical‐to‐basolateral Cl^−^ gradient and 10 *μ*mol/L forskolin was added to stimulate CFTR. (D) summary of HCO_3_^−^ secretion rates under each condition (mean ± SEM, *n* = 4–9). HCO_3_^−^ secretion by Calu‐3 Scr‐KD and PDS‐KD monolayers was negligible in unstimulated monolayers with Cl^−^ gradient, and similar after forskolin addition (ns, not significant, *P* > 0.2).

To investigate anion exchange independently of the basolateral membrane, monolayers were mounted in Ussing chambers, bathed symmetrically with nominally Cl^−^‐free solution, and the basolateral membrane was permeabilized by adding 360 *μ*g·mL^−1^ nystatin. The appearance of HCO_3_^−^ on the apical side was monitored using pH‐stat when an apical‐to‐basolateral Cl^−^ gradient was imposed by apical addition of 30 mmol·L^−1^ NaCl. We showed previously that the small osmotic gradient that results from asymmetrical addition of NaCl does not affect *I*_eq_ or HCO_3_^−^ flux significantly (Shan et al. 2012). The Cl^−^ gradient produced a small inward current as expected with basal Cl^−^ conductance. Forskolin stimulated a much larger negative *I*_eq_ across parental, Scr‐KD, and PDS‐KD Calu‐3 monolayers, consistent with activation of CFTR channels in the presence of an apical‐to‐basolateral Cl^−^ gradient (Fig. 4A–C). We showed previously that the forskolin‐stimulated negative *I*_eq_ measured under these conditions is abolished in CFTR‐KD cells (Shan et al. 2012). No HCO_3_^−^ flow in the opposite (secretory) direction was detected under basal conditions but it appeared after forskolin was added, suggesting efflux through CFTR. The outward HCO_3_^−^ fluxes were similar in parental Calu‐3 cells (0.554 ± 0.049 *μ*equiv·cm^−2^·h^−1^, *n* = 6, *P* > 0.2), Scr‐KD (0.414 ± 0.037 *μ*equiv·cm^−2^·h^−1^, *n* = 5), and PDS‐KD (PDS‐KD: 0.369 ± 0.057 *μ*equiv·cm^−2^·h^−1^, *n* = 9) indicating that they are independent of pendrin (Fig. 4D).

### Effect on pH_i_ of removing extracellular Cl^−^

**Figure 5:**
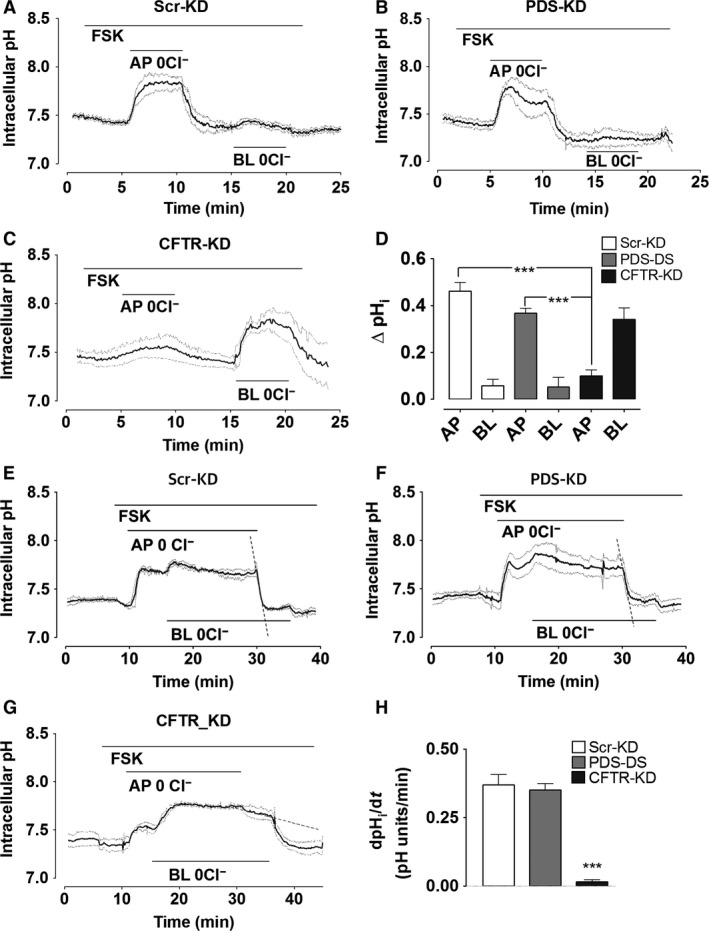
pH_i_ responses to extracellular Cl^−^ substitution. Calu‐3 monolayers were stimulated with forskolin in HCO_3_^−^‐buffered solution and Cl^−^ was replaced with gluconate on the apical, basolateral or both sides as indicated by horizontal bars. Continuous traces in each show the mean pH_i_ and dashed traces show ± SEM for all experiments (*n* = 4–5). (A) Scr‐KD cells, (B) PDS‐KD cells, (C) CFTR‐KD cells. (D) mean change in pH_i_ induced by Cl^−^ substitution on the apical (AP) or basolateral (BL) side. Alkalinizations were similar in Scr‐KD and PDS‐KD cells (means ± SEM, *n* = 4–5, *P* > 0.2) but greatly reduced in CFTR‐KD cells (***P < 0.001). (E) initial rate of re‐acidification (dashed line) when 124 mmol/L Cl^−^ was restored on the apical side of forskolin‐stimulated monolayers (after bilateral Cl^−^‐free perfusion for >15 min to eliminate basolateral exchange). Apparent anion exchange at the apical membrane was similar in (E) control Scr‐KD and (F) PDS‐KD cells, but greatly reduced in CFTR‐KD cells (G). (H) summary of reacidification rates in different cell lines challenged with apical low‐Cl^−^ solution.

Fluorescence imaging was also used to study apical anion exchange. pH_i_ was measured when cells were challenged with nominally Cl^−^‐free apical solution containing 25 mmol/L HCO_3_^−^. Cl^−^ removal from the apical side during forskolin stimulation increased pH_i_ in Scr‐KD cells (ΔpH_i_ = 0.46 ± 0.04 units, *n* = 4) consistent with inward HCO_3_^−^ flow through activated CFTR channels, whereas basolateral Cl^−^ removal had little effect (0.06 ± 0.03 units, *n* = 5, *P* = 0.0016; Fig. 5A). To test if the alkalinization requires pendrin or CFTR, Scr‐KD cell pH_i_ responses were compared with those obtained using PDS‐KD (Fig. 5B) and CFTR‐KD (Fig. 5C) cells. Forskolin‐stimulated PDS‐KD cells showed a similar large increase in pH_i_ during challenge with low apical Cl^−^ (0.38 ± 0.05 pH unit, *n* = 4; Fig. 5B) and there was no change in pH_i_ upon removal of basolateral Cl^−^ (0.05 ± 0.04 pH unit, *n* = 4, *P* = 0.0055). The inability of basolateral Cl^−^ removal to alkalinize Scr‐KD or PDS‐KD cells despite the presence of basolateral AE2 anion exchangers would be explained if the HCO_3_^−^ that is taken up by basolateral exchange escapes through apical CFTR channels during forskolin stimulation (Kim et al. 2014). To test that interpretation we also measured pH_i_ responses during basolateral Cl^−^ removal in CFTR‐KD cells and forskolin stimulation (Fig. 5C). In CFTR‐deficient cells, intracellular alkalinization induced by apical Cl^−^ substitution was attenuated (0.1 ± 0.02 pH unit, *n* = 4; Fig. 5C) while that produced by basolateral Cl^−^ removal was greatly increased (0.34 ± 0.05 pH unit, *n* = 4, *P* = 0.042). Figure 5D summarizes these pH_i_ responses to extracellular Cl^−^ removal in all three cell lines. We conclude that forskolin normally prevents pH_i_ alkalinization during exposure to low basolateral Cl^−^ because basolateral HCO_3_^−^ taken up by the cells exits through apical CFTR channels.

Apical HCO_3_^−^/Cl^−^ exchange could potentially be missed due to HCO_3_^−^ efflux through basolateral AE2 exchangers. To test this possibility, intracellular Cl^−^ was depleted by Cl^−^ removal from both sides for ≥15 min, then Cl^−^ was restored only on the apical side while anion exchange‐mediated apical HCO_3_^−^ efflux was monitored as the rate of re‐acidification (Fig. 5E–H). Under these conditions, apical HCO_3_^−^ efflux was similar in Scr‐KD (Fig. 5E; dashed line, 0.37 ± 0.03 pH units min^−1^) and PDS‐KD cells (Fig. 5F; dashed line, 0.35 ± 0.04 pH units min^−1^, *n* = 4) whereas a much smaller and slower reacidification was observed upon restoration of Cl^−^ to the apical side of CFTR‐KD cells (0.015 ± 0.01 pH units min^−1^, dashed line, *n* = 4, Fig. 5G). These results, which are summarized in Figure 5H, provide further evidence that CFTR is the predominant apical pathway for HCO_3_^−^ in Calu‐3 cells and that pH_i_ responses to apical Cl^−^ removal are due to the Cl^−^ efflux that is electrically coupled to HCO_3_^−^ entry through CFTR channels.

### Pharmacological inhibitor effects on pH_i_

**Figure 6:**
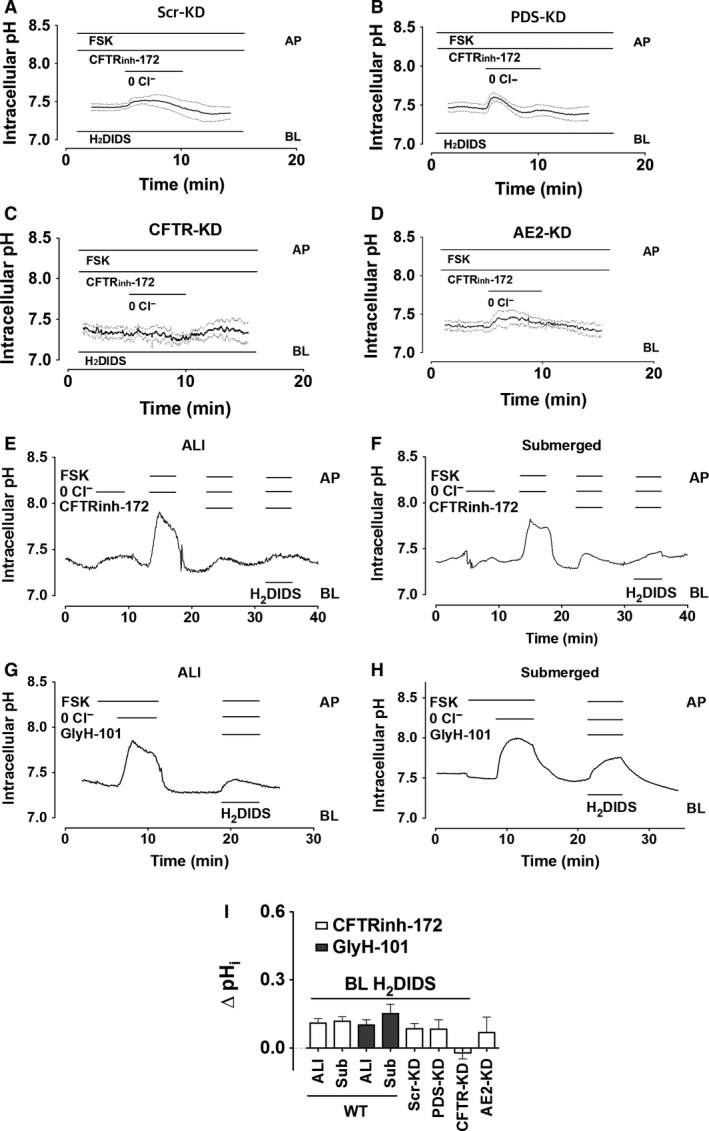
Anion exchange in cAMP‐stimulated Calu‐3 monolayers exposed to basolateral H_2_DIDS and apical CFTR_inh_‐172. Polarized cells were superfused with HCO_3_^−^‐buffered solution and apical Cl^−^ was replaced with gluconate during stimulation with 10 *μ*mol/L forskolin and exposure to basolateral 500 *μ*mol/L H_2_DIDS as indicated. Continuous traces show the mean pH_i_ and the dashed lines indicate ± SEM, *n* = 4–7. (A) SCR‐KD (control) cells, (B) Pendrin‐KD cells, (C) CFTR‐KD cells, and (D) AE2‐ KD cells. (I) summary of alterations in pH_i_ induced by apical Cl^−^ substitution using parental (WT) Calu‐3 cells that had been cultured under air–liquid interface (ALI) or submerged (Sub) conditions and exposed acutely to CFTR inhibitors on the apical side and to basolateral H_2_DIDS to minimize HCO_3_^−^ flux through non‐pendrin transporters. Also shown are the responses to apical Cl^−^ substitution together with basolateral H_2_DIDS from experiments in (A–D) using Scr‐KD (control) and knockdown cell lines deficient in PDS, CFTR, and AE2.

A large alkalinization (0.8 pH unit) has been reported during apical Cl^−^ removal while apical CFTR and basolateral HCO_3_^−^ transport are both inhibited pharmacologically, and this provided strong evidence for pendrin‐mediated HCO_3_^−^ transport (Garnett et al. 2011). We performed similar experiments using both control and pendrin knockdown cell lines (Fig. 6A–C). pH_i_ was measured in unstimulated cells while they were challenged with nominally Cl^−^‐free apical solution and continuously exposed to basolateral H_2_DIDS (500 *μ*mol/L; to inhibit HCO_3_^−^ flux through AE2 and Na^+^‐ HCO_3_^−^ cotransporters) and apical CFTR_inh_‐172 (10 *μ*mol/L; to inhibit HCO_3_^−^ flux through CFTR).

As expected, removing apical Cl^−^ under these conditions caused a small alkalinization in Scr‐KD and PDS‐KD cells (ΔpH_i_ = 0.09 ± 0.02 and 0.08 ± 0.04 pH units·min^−1^, respectively, *n* = 4; Fig. 6A and B), about 25% of that observed without inhibitors present (compare with Fig. 5 and B). Basal CFTR activity was apparently responsible for the intracellular alkalinization because it was further reduced in CFTR‐KD cells. Similar results were obtained using AE2‐KD cells (Huang et al. 2012) that were exposed to CFTR_inh_‐172 (ΔpH_i_ = −0.02 ± 0.03, 0.06 ± 0.07 pH units·min^−1^, *n* = 6–7, respectively, Fig. 6C and D). Thus, pH_i_ is not increased significantly by low apical [Cl^−^] in any of the cell lines when CFTR and basolateral anion exchangers are inhibited. These results differ from those reported previously using Calu‐3 cells that had been cultured under submerged conditions rather than at the ALI, therefore the experiments were repeated using cells that had been cultured under submerged conditions. When parental Calu‐3 cells were stimulated using forskolin to activate CFTR, robust alkalinization was observed during apical Cl^−^‐free exposure regardless of whether they were cultured at the ALI (Fig. 6G) or submerged (6H); compare with Figures 6E and F, respectively. These responses were inhibited by CFTR_inh_‐172 or GlyH‐101 and were not restored by inhibiting potential basolateral HCO_3_^−^ efflux with H_2_DIDS (Fig. 6I). We conclude from these inhibitor studies that the apparent anion exchange at the apical membrane is mediated by electrical coupling in CFTR channels and is similar whether cells are cultured under ALI or submerged conditions.

### Dependence of pH_i_ on apical HCO_3_^−^ concentration

Exposure to nominally Cl^−^‐free solutions may alter cell volume and have other effects that are unrelated to anion exchange, therefore we also examined pendrin‐dependent anion exchange by measuring the pH_i_ response to variations in apical [HCO_3_^−^]. Another advantage of this protocol is that extracellular [Cl^−^] remains constant and HCO_3_^−^ efflux is not limited by the supply of intracellular Cl^−^ when extracellular [HCO_3_^−^] is lowered, thereby avoiding dependence on apical Cl^−^ recycling through CFTR.

**Figure 7:**
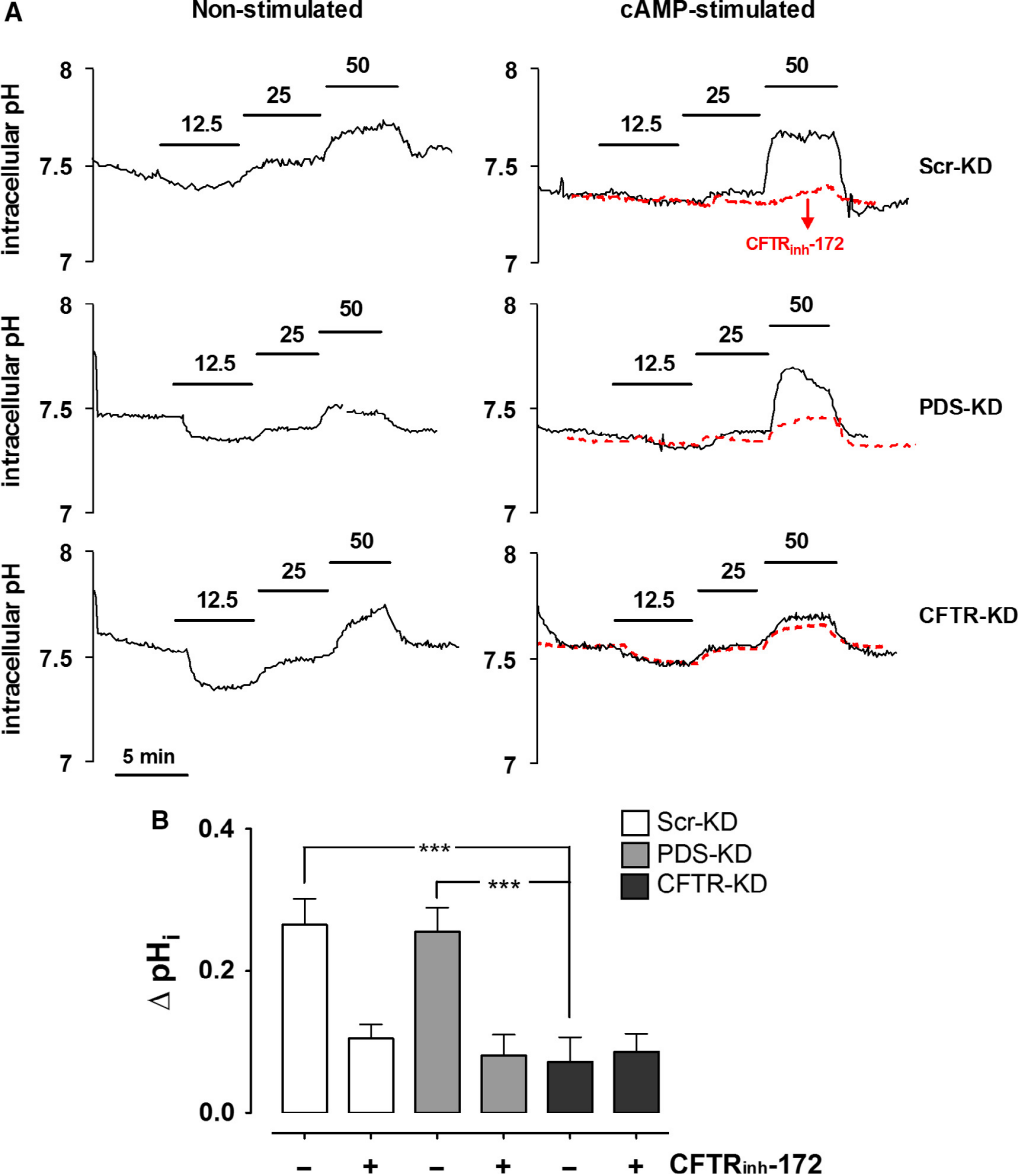
Effects of altering apical HCO_3_^−^ concentration on pH_i_ in control, pendrin knockdown, and CFTR knockdown Calu‐3 cell lines. (A) intracellular pH was measured in Scr‐KD, PDS‐KD, and CFTR‐KD monolayers bathed basolaterally with HCO_3_^−^‐buffered solution (25 mmol/L) and H_2_‐DIDS, and challenged with the apical [HCO_3_^−^] shown. Recordings on the left were obtained without forskolin stimulation, those on the right with forskolin. Also shown with red dashed lines are the mean responses when 10 *μ*mol/L CFTR_inh_‐172 was added to assess the dependence on CFTR under these conditions. (B) summary of pH_i_ changes in stimulated cells induced by exposure to high apical [HCO_3_^−^] (50 mmol/L) in the absence (−) or presence (+) of CFTR_inh_‐172 (mean ± SEM, *n* = 4; ***P < 0.001).

Apical HCO_3_^−^ concentration was varied as follows: it was reduced twofold by isosmolal replacement with gluconate, then after a control period with 25 mmol/L [HCO_3_^−^], its concentration was increased twofold by the addition of NaHCO_3_. DIDS (100 *μ*mol/L) was present on the basolateral side throughout to inhibit basolateral HCO_3_^−^ flux through AE2 or NBC. As shown in Figure 7A (traces on the left side), decreasing apical [HCO_3_^−^] to 12.5 mmol/L reduced pH_i_, however, this response was variable and not different between the three cell lines. Increasing [HCO_3_^−^] to 50 mmol/L produced a small, reversible alkalinization. Similar results were obtained using Scr‐KD, PDS‐KD, and CFTR‐KD cell lines (ΔpH_i_ = 0.1 ± 0.06, 0.09 ± 0.02, and 0.11 ± 0.05 pH units·min^−1^, respectively, *n* = 4). During forskolin stimulation (Fig. 7A, traces on the right side), the alkalinization induced by 50 mmol/L HCO_3_^−^ was enhanced by forskolin in Scr‐KD and PDS‐KD cells (ΔpH_i_ = 0.27 ± 0.04, 0.26 ± 0.03 pH units·min^−1^, respectively) but not in CFTR‐KD cells (0.07 ± 0.07 pH units·min^−1^), indicating that the apical HCO_3_^−^ entry that leads to alkalinization requires CFTR but not pendrin. Further evidence for CFTR‐mediated HCO_3_^−^ influx under these apical 50 mmol/L [HCO_3_^−^] conditions came from the inhibitory effect of CFTR_inh_‐172 on the intracellular alkalinizations in both Scr‐KD and PDS‐KD cells (10 *μ*mole·L^−1^; red dashed lines in Fig. 7A). As summarized in Figure 7B, these results with knock down cell lines and CFTR_inh_‐172 indicate that most HCO_3_^−^ flows through CFTR.

### IL‐4 does not increase pendrin expression or secretion by Calu‐3

**Figure 8:**
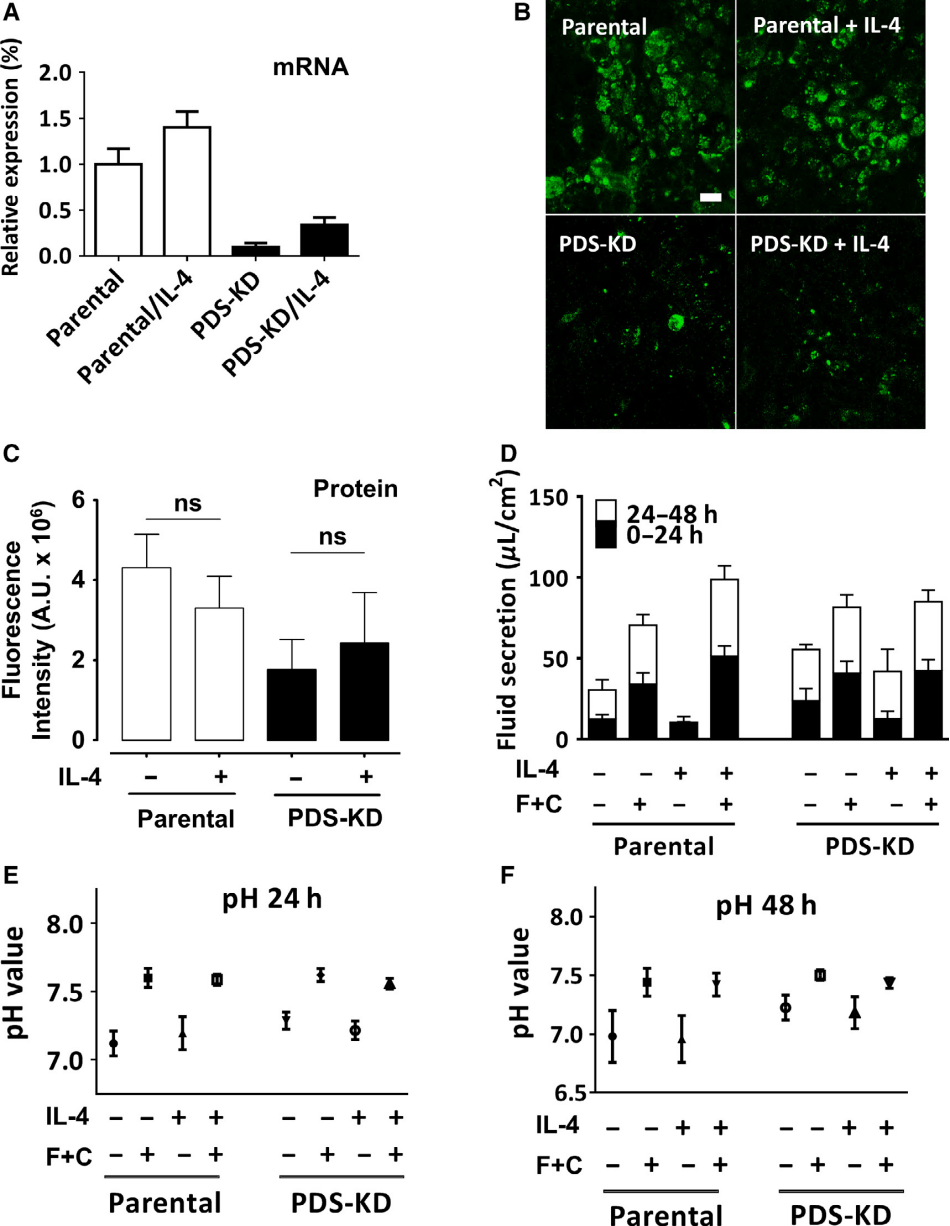
IL‐4 effects on pendrin expression in Calu‐3 cells, fluid secretion rate, and pH of secretions. (A) relative pendrin mRNA expression in cell lines measured using quantitative real‐time PCR and normalized to GAPDH after 48 h treatment with IL‐4 (10 ng/mL) at the air–liquid interface. (B) background‐subtracted images of parental (WT) and pendrin knockdown (PDS‐KD) cells immunostained for pendrin with or without 48 h pretreatment with IL‐4 (10 ng/mL). (C) summary of image fluorescence intensities in arbitrary units (A.U.). ns, not significant. (*P* > 0.05). Pendrin protein staining was not increased significantly in Calu‐3 cells treated with IL‐4. (D) cumulative fluid secretion after 2 day pretreatment with DMSO (vehicle) or IL‐4 (10 ng/mL). Fluid was collected at 24 h intervals. cAMP + forskolin (C+F; or vehicle control) was added in some experiments to activate CFTR. (E and F) pH of the fluid secreted in panels (C) 0–24 h, and (D) 24–48 h, respectively.

Pendrin expression is strongly upregulated in primary surface airway epithelial cells during inflammation therefore we studied Calu‐3 cells exposed to the cytokine IL‐4 (10 ng·mL^−1^) for 48 h. qPCR revealed a slight increase in pendrin mRNA which was not statistically significant (*P* = 0.18, *n* = 3, Fig. 8A). Likewise, pendrin protein level was not increased significantly after IL‐4 treatment in scr‐KD control (13.1 ± 15.2%, ns, *n* = 5) or PDS‐KD (12.9 ± 16.1%, ns, *n* = 5) cell lines according to immunostaining (Fig. 8B and C). IL‐4 alone did not alter fluid secretion by parental Calu‐3 monolayers, however, there was a trend toward higher forskolin‐stimulated secretion that did not reach statistical significance at *P* < 0.05 (but did at *P* < 0.1, Fig. 8D; *n* = 4, *P* = 0.08). The apparent increase in forskolin‐stimulated fluid secretion after IL‐4 was not statistically significant in parental cells and was completely absent in PDS‐KD cells. Forskolin increased the pH of secretions by ~0.5 pH units regardless of the presence of IL‐4 (Fig. 8E and F). In summary, IL‐4 did not increase pendrin expression significantly in Calu‐3 cells and had little effect on the secretion of fluid and HCO_3_^−^, and pendrin knockdown did not alter the pH or volume of secretions (*n* = 6–9, *P* > 0.2).

### Effect of culture conditions on anion transporter expression

**Figure 9:**
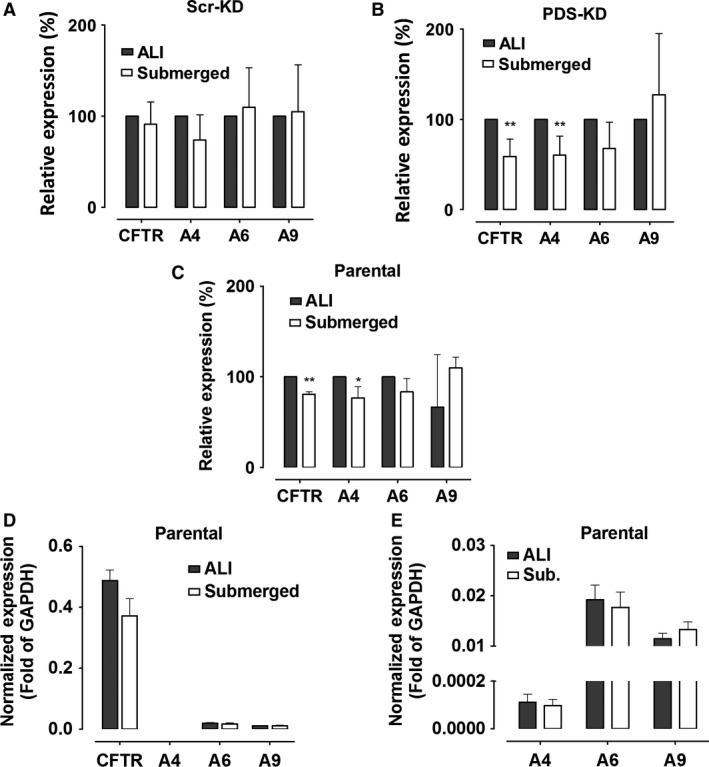
Comparison of CFTR, pendrin, SLC26A6, and SLC26A9 expression in cultures at the air–liquid interface (ALI) versus submerged conditions. qRT‐PCR was performed using (A) Scr‐KD (control) cells, (B) PDS‐KD, and (C) parental cells (WT), normalized to the levels in ALI cultures. (mean ± SEM, *n* = 3–5; *P < 0.05, **P < 0.01, one‐tailed Student's *t* test). (D) comparison of qRT‐PCR results for the four genes examined, each normalized to GAPDH expression, showing relative levels of CFTR and SLC26A transporters. (E) relative expression of SLC26A transporters, rescaled to enable comparison. Note that after normalization to GAPDH, the qRT‐PCR signals for SLC26A6 and SLC26A9 were >100‐fold higher than for pendrin.

Abnormally low pendrin expression or high CFTR expression under our ALI conditions could potentially explain the negligible role of pendrin in this study, therefore we compared cells that had been cultured under ALI versus submerged conditions, which are known to impact Calu‐3 differentiation (Kreft et al. 2015). Figure 9A–C shows that ALI and submerged cultures had similar transcript levels for all four genes in control Scr‐KD cells, PDS‐KD cells, and in low‐passage parental cells from ATCC. Levels of CFTR, SLC26A4, SLC26A6, and SLC26A9 mRNA under submerged conditions are shown relative those in ALI cultures after normalization to GAPDH (Fig. 9D and E). Although the levels of different transcripts cannot be rigorously compared, the results suggest that expression of SLC26A transporters is weak relative to CFTR and to other SLC26A family members, with levels of SLC26A6 and SLC26A9 transcripts being ~100‐fold higher than those of pendrin. The functions of SLC26A6 and SLC26A9 in Calu‐3 cells remain to be determined.

## Discussion

In this study we have examined apical anion transport in the Calu‐3 cell line using converging approaches and found that CFTR is the predominant pathway for HCO_3_^−^ efflux. Pendrin mRNA was expressed in Calu‐3 cells as expected (Garnett et al. 2011) but its levels were low compared to both CFTR and the basolateral anion exchanger AE2, in agreement with a previous study (Kim et al. 2014), and knocking down pendrin did not alter HCO_3_^−^ secretion. Pendrin protein was not detected reliably on immunoblots but was clearly observed by immunostaining as reported previously (Garnett et al. 2011). Pendrin mRNA and immunofluorescence were both reduced ~80% by shRNA stably expressed using a lentivirus. The proinflammatory Th2 cytokine IL‐4, which strongly induces pendrin expression in bronchial epithelial cells (Galietta et al. 2002), did not increase pendrin mRNA levels significantly in marked contrast to surface airway cells. The Calu‐3 cell line may lack some molecule in the IL‐4 receptor signaling pathway which is present in surface airway epithelia and essential for upregulating pendrin expression. Thus our conclusion regarding the lack of pendrin‐mediated bicarbonate secretion is restricted to the Calu‐3 cell line. It is reasonable to expect pendrin to mediate significant bicarbonate flux in surface airway epithelial cells especially when upregulated by cytokines, as has been reported by others (Gorrieri et al. 2016).

We compared anion and fluid transport by control and pendrin knock down cells and found no evidence for pendrin‐dependent anion exchange activity using pH‐stat or fluorescence assays of pH_i_. We studied apical Cl^−^/HCO_3_^−^ exchange with pH‐stat by imposing an apical‐to‐basolateral Cl^−^ gradient and permeabilizing the basolateral membrane. Net HCO_3_^−^ secretion was only detected under these conditions if forskolin was added, and the HCO_3_^−^ flux was similar in control and pendrin knockdown cells suggesting it occurs through CFTR. Control and pendrin knockdown cells had similar rates of intracellular reacidification when cells were HCO_3_^−^ loaded by exposure to apical low‐Cl^−^ solution. Although at first glance this might seem to be evidence for apical anion exchange, inhibitor studies and the effects on pH_i_ of manipulating extracellular HCO_3_^−^ were consistent with CFTR‐mediated HCO_3_^−^ flux. Together the results suggest that opposing Cl^−^ and HCO_3_^−^ fluxes are coupled electrically in the CFTR channel pore rather than chemically through pendrin‐mediated Cl^−^/HCO_3_^−^ exchange (Kim et al. 2014). We cannot exclude the possibility that some CFTR‐dependent HCO_3_^−^ flux occurs through another type of anion channel that may be regulated by CFTR such as SLC26A9 (Bertrand et al. 2009), however, sensitivity to both CFTR_inh_‐172 and GlyH‐101 suggests that most HCO_3_^−^ flux is mediated by CFTR channels.

The present results differ from a previous study in which pendrin knockdown caused a reduction in the pH of secretions whereas silencing CFTR had no effect (Garnett et al. 2011). The reason for the discrepancy is not known, however, different cell lines were used in the two studies. The extent of pendrin knockdown was probably similar in the two studies as residual pendrin mRNA expression was 8.5% in cells used previously (Garnett et al. 2011) versus 10.7 ± 9% in the cells used here. CFTR knock down was less complete in the previous study (28 ± 5% residual CFTR) compared to the cell line used here (~5% residual CFTR) which might reduce the apparent role of CFTR (Palmer et al. 2006a; MacVinish et al. 2007; Garnett et al. 2011). Another technical difference was our use of air–liquid interface culture whereas submerged cultures were used previously. However, we found here that this had little effect on relative anion transporter expression (Fig. 9) or apical anion exchange activity (Fig. 6E–H), thus further work is needed to reconcile the results of these studies.

Replacing basolateral Cl^−^ with gluconate increased pH_i_ in all Calu‐3 cell lines examined. This was anticipated since there is robust AE2 mediated Cl^−^/HCO_3_^−^ exchange at the basolateral membrane (Loffing et al. 2000; Huang et al. 2012). However, the alkalinization observed during forskolin stimulation was >6‐fold larger in CFTR‐KD cells than in Scr‐KD (control) and PDS‐KD cells. This strongly suggests that intracellular HCO_3_^−^ accumulates during exposure to basolateral low‐Cl^−^ solution as long as apical CFTR is not functional, presumably because HCO_3_^−^ taken up escapes through apical CFTR channels when they are activated by forskolin.

In summary, the present results indicate that most HCO_3_^−^ secretion by Calu‐3 cells occurs via CFTR (Shan et al. 2012; Kim et al. 2014). This is consistent with the HCO_3_^−^ permeability of CFTR channels (Gray et al. 1990; Poulsen et al. 1994; Linsdell et al. 1997) and the large contribution of CFTR to apical membrane conductance in Calu‐3 cells (Tamada et al. 2001). Pendrin may play a more important role in HCO_3_^−^ secretion by surface airway epithelial cells where it is strongly upregulated by the proinflammatory cytokine IL‐4 (Gorrieri et al. 2016).

## Conflict of Interest

The authors declare they have no conflicts of interest with the contents of this article.
